# Differential Metabolomics and Cardiac Function in Trained vs. Untrained Yili Performance Horses

**DOI:** 10.3390/ani15162444

**Published:** 2025-08-20

**Authors:** Tongliang Wang, Jun Meng, Xixi Yang, Yaqi Zeng, Xinkui Yao, Wanlu Ren

**Affiliations:** 1 College of Animal Science, Xinjiang Agricultural University, Urumqi 830052, China; wtl13639911402@163.com (T.W.); mengjun@xjau.edu.cn (J.M.); xxyang2022@126.com (X.Y.); zengyaqi@xjau.edu.cn (Y.Z.); yaoxinkui@xjau.edu.cn (X.Y.); 2 Xinjiang Key Laboratory of Horse Breeding and Exercise Physiology, Urumqi 830052, China; 3 Horse Industry Research Institute, Xinjiang Agricultural University, Urumqi 830052, China

**Keywords:** Yili horses, echocardiography, broad-targeted metabolomics, exercise performance, training

## Abstract

This study aimed to investigate the effects of training on cardiac structure and function, as well as plasma metabolite profiles in horses, in order to uncover the molecular regulatory mechanisms and cardiac remodeling under long-term exercise. We hypothesize that long-term standardized training induces physiological cardiac remodeling and differential metabolomic changes in Yili horses, which correlate with improved athletic performance. The study focuses on physiological exercise-induced cardiac remodeling, characterized by increased left ventricular wall thickness and chamber size. A total of 18 Yili horses, a unique Chinese equine breed, were included in the study of equine exercise physiology. Twelve horses underwent six months of standardized training followed by three 1000 m performance tests. Based on final rankings, they were divided into an advanced group (AG, top six horses) and a habitual group (HG, bottom six horses). The remaining six untrained horses served as the untrained group (UG), with only free-range activity. Echocardiographic results revealed significant differences (*p* < 0.05) between the trained and untrained groups in cardiac parameters such as LVID, LVFW, LVM, AODd, IVSs, HR, EDV, ESV, LADs, LVLD, MVD, PADs, and SV. Further comparison between AG and HG showed significant differences in AODd, EESV, HR, IVSd, LVIDs, LVM, RVDd, and RVDs (*p* < 0.05). Metabolomic analysis identified 465 differential metabolites between AG and HG, 456 between AG and UG, and 379 between HG and UG, with 106 overlapping metabolites among all three groups. Plasma metabolomics revealed significant negative correlations between specific long-chain lysophosphatidylcholines (LPCs) and cardiac structural parameters (LVIDd, LVFWD, LVIDs, LVLD, MVD, and LADs), whereas LPC (O-18:2) showed an opposite trend. Key metabolites such as 3-hydroxybutanoic acid, carnitine C4:0, carnitine isoC4:0, hippuric acid, and uric acid were significantly lower in AG compared to HG and UG, with uric acid levels negatively correlated with LVID and LVM. Glycerophospholipid metabolism emerged as the core pathway differentiating exercise capacity among all groups. Notably, efferocytosis (vs. HG and UG) and tryptophan metabolism/aromatic amino acid biosynthesis (vs. HG) were specifically enriched in AG. These findings provide a novel theoretical basis and research perspective for optimizing racehorse training strategies and exploring the metabolic regulation of the athletic heart.

## Simple Summary

Training racehorses effectively requires understanding how exercise improves their bodies. This study examined Yili horses, a unique Chinese breed known for speed and stamina. We compared heart structure and blood chemistry in the following three groups: habitual group, advanced group, and untrained horses. Heart scans revealed that trained horses developed thicker heart walls and larger pumping chambers, especially in the left ventricle—key adaptations for better performance. Blood tests identified specific metabolic changes linked to training, particularly in fats called glycerophospholipids and energy-related molecules like 3-hydroxybutyric acid. These changes help the heart and muscles use fuel more efficiently during exercise. Importantly, blood markers like lysophosphatidylcholine (LPC) and carnitine levels strongly correlated with heart structure and performance. These findings enhance our understanding of how long-term training affects horses with different athletic capacities as well as untrained horses and provide a potential method for identifying athletic potential through blood biomarkers in combination with echocardiographic evaluation.

## 1. Introduction

The Yili horse performance test is one of the most popular equestrian events in Xinjiang, China. Currently, the number of Yili horses registered with the Xinjiang Horse Industry Association has reached 15,000. Yili horses are robust, well-proportioned, fast, and have excellent endurance. They are China’s first sport horse with independent intellectual property rights, and their performance has been continuously improving in recent years [1].

The heart is a crucial part of the circulatory system, responsible for continuously delivering oxygen, water, inorganic salts, proteins, glucose, and various water-soluble vitamins to all organs of the body through the blood [2]. Echocardiography is a non-invasive technique widely used to evaluate the anatomical and functional status of the heart and large vessels in sport horses. Research on the correlation between equine sports performance and cardiovascular function is extensive, covering areas such as disease diagnosis, auxiliary treatment, the impact of exercise, and health evaluation [3,4,5,6]. Studies have shown that exercise training induces changes in heart structure and function, and different types of events also affect the heart structure differently. Left ventricular internal diameter was highly associated with endurance-type, long-duration high-intensity exercise, whereas left ventricular wall thickness was significantly related to short-duration, high-intensity activities such as show jumping [3]. However, factors such as breed, age, gender, and weight also influence heart function [4], and research findings from other horse breeds cannot necessarily be applied to Yili horses.

Studies have shown that long-term training leads to higher levels of branched-chain amino acid (BCAA)-derived acylcarnitines and diacylglycerols (DAGs) in the muscles of trained horses compared to untrained horses. Training also increases lipid and protein metabolism and the concentrations of total essential amino acids (EAAs) and BCAAs in plasma [5]. In a study involving 32 healthy, well-trained, thoroughbred horses, high-intensity exercise was found to increase the content of esterified L-carnitine in plasma [6]. Additionally, studies comparing pre- and post-race metabolic profiles revealed that metabolites involved in glucose, lipid metabolism, ketone bodies, and acetic acid could predict the probability of race completion [7].

Most of the studies in recent years have focused on specific training methods or the short-term effects before and after the competition. However, there is still a lack of systematic comparisons between the well-trained group, the average-trained group, and the non-trained group after long-term training. The focus of this study was to understand how cardiac structure and function, as determined by echocardiography, were associated with enhanced performance levels in Yili racehorses and how differential expression of specific metabolites affected exercise-mediated cardiac remodeling and strengthening of the cardiovascular system. By using an integrated bioinformatics approach, this study aimed to provide valuable insights into how training can be designed to improve cardiac health and performance in Yili horses.

## 2. Materials and Methods

### 2.1. Ethics Statement

The experimental procedures adhered to the ARRIVE guidelines and were approved by the Animal Welfare Committee of Xinjiang Agricultural University (approval no. 2023037). The owners of all horses provided informed consent for the experiments.

### 2.2. Animals

A total of 18 two-year-old Yili horses (9 males and 9 females) with minimal differences in body structure (Table S1) were selected for the study; feeding and management conditions were consistent with accepted husbandry standards. Although all horses were two years old and in their growth phase, body structure differences were minimized, and an untrained control group of the same age was included to differentiate training effects from growth-related changes. All horses underwent health checks, including cardiovascular and respiratory system examination and routine blood tests. Twelve horses underwent six months of training (see Supplementary Text S1: Training plan) at a consistent intensity. The heart rate of the horses and exercise intensity were monitored using a heart rate sensor with heart rate straps. As controls, six horses were not trained and were allowed to move freely around the paddock.

After completing the training period, the horses participated in three 1000 m performance trials (Table S2). Based on the average race time, the top six finishers were categorized as the advanced group (AG, 75.7 ± 1.56 s), the bottom six as the habitual group (HG, 80.24 ± 2.36 s), and the free-moving group as the untrained control group (UG).

### 2.3. Echocardiography and Blood Sample Collection

Cardiac structure and function were assessed using non-invasive echocardiography. No electrophysiological or biopsy analyses were performed. The horses were restrained in stocks to limit their range of movement, thereby preventing interference with body measurements and echocardiographic data collection. The examination area was cleaned with water and alcohol, and an ultrasound probe was used on the right side of the horse between the third and fourth ribs. The frequency was set to 2.5 MHz, and the angle was adjusted to 110°. Three B-mode images and dynamic images were captured from the right parasternal long-axis, right parasternal short-axis, and the right parasternal left ventricular outflow tract. Echocardiographic images were captured at rest within a heart rate range of 35–50 bpm (Polar H10). Measurements corresponded to specific cardiac cycle phases (end-diastole and end-systole). An average value was calculated from three readings. For plasma collection, a 5 mL blood sample was drawn from the jugular vein into an EDTA tube while the horse was at rest. The blood was centrifuged at 3443× *g* for 10 min, and the plasma was collected, aliquoted, and stored in liquid nitrogen. All samples were submitted for analysis together at the end of the experiment.

**Figure 1:**
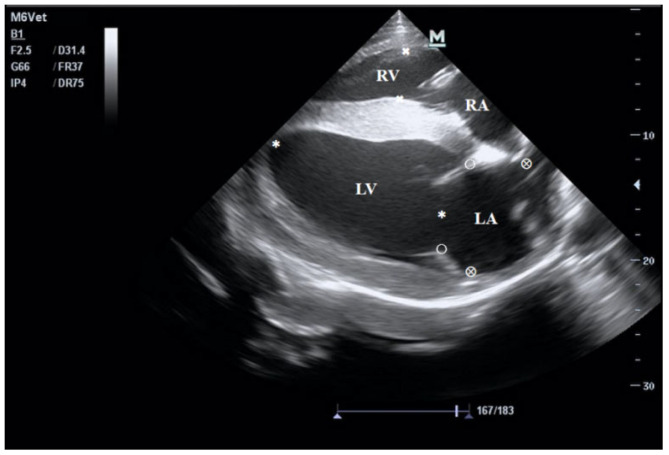
B-mode echocardiogram of the right parasternal long-axis four-chamber view. LV: left ventricular; RV: right ventricular; RA: right atrium; LA: left atrial.

**Figure 2:**
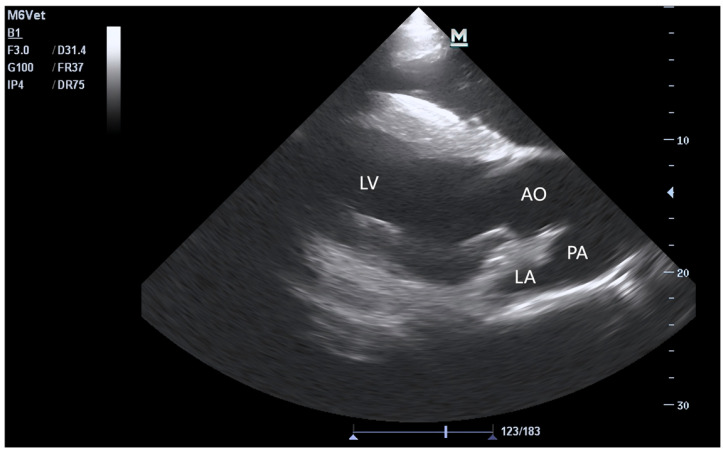
B-mode echocardiogram of the right parasternal long-axis view of the left ventricular outflow tract. PA: pulmonary artery, AO: aorta.

**Figure 3:**
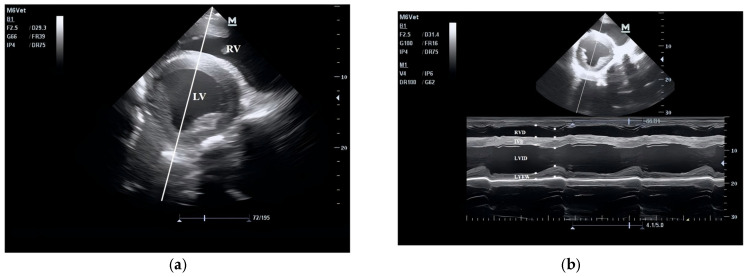
B-mode (**a**) and M-mode (**b**) echocardiogram of the right parasternal short-axis view.

**Figure 4:**
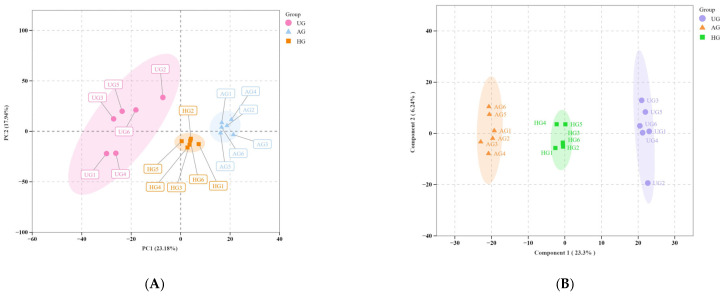
(**A**) PCA plots. PC1 represents the first principal component and PC2 represents the second principal component, and the percentage indicates the proportion of the data set explained by PC1 and PC2. Each point in the figure represents a sample, and samples from the same group are shown in the same color. Group refers to the grouping. (**B**) OPLS-DA score plots. The horizontal axis represents the predicted principal component, and the direction of the horizontal axis shows the gap between groups. The vertical axis represents the orthogonal principal component, and the direction of the vertical axis shows the gap within groups. The percentage indicates the interpretation rate of this component for the data set. Each point in the figure represents a sample, and samples within the same group are shown in the same color. “Group” indicates the grouping.

The echocardiograms of the three groups at rest are shown in Figure 1, Figure 2, Figure 3 and Figure 4, including the right parasternal long-axis, four-chamber B-mode echocardiogram; right parasternal long-axis, left ventricular outflow tract, B-mode echocardiogram, right parasternal short-axis, M-mode echocardiogram, and right parasternal short-axis B-mode echocardiogram. The corresponding measurements are indicated in the figures. Detailed parameters are listed in Table 1. The echocardiographic parameters before training are shown in Table S3. Since UG horses did not undergo training, they did not participate in the 1000 m test races.

The echocardiogram was obtained at the fourth intercostal space on the right side, with the transducer pointing toward the fifth intercostal space on the left side. Measurements included the left ventricular long diameter (LVLD, ✱), right ventricular diameter (RVD, ×), mitral valve diameter (MVD, ○), and left atrial diameter (LAD, ⊗). RV: right ventricle; LA: left atrium; LV: left ventricle.

The echocardiogram was taken at the fourth intercostal space on the right side, and the transducer points to the fourth intercostal space on the left side.

Images were obtained at the mid-papillary muscle level from the fourth intercostal space on the right side, with the probe pointing towards the abdomen. The baseline was obtained on the B-mode echocardiogram of the left ventricle (Figure 4A), and this line indicates the cursor position for obtaining the M-mode echocardiogram of the left ventricle at the mid-sagittal level (Figure 4B). The right ventricular internal diameter (RVD), interventricular septum (IVS), left ventricular internal diameter (LVID), and left ventricular free wall (LVFW) were measured. The end-diastolic (✱) point is the point where the left ventricle relaxes to its maximum and is about to start contracting, and the end-systolic (×) point is the maximum deflection point of the interventricular septum.

$$\mathrm{EF}\%=\frac{\mathrm{EDV}\left(\mathrm{mL}\right)-\mathrm{ESV}\left(\mathrm{mL}\right)}{\mathrm{EDV}\left(\mathrm{mL}\right)}$$

$$\mathrm{FS}\%=\frac{\left(\mathrm{LVIDd}-\mathrm{LVIDs}\right)\times 100}{\mathrm{LVIDd}}$$

$$\mathrm{LVM}(g)=1.04\times \left[{\left(\mathrm{LVIDd}+\mathrm{LVFWd}+\mathrm{IVSd}\right)}^{3}-{\mathrm{LVIDd}}^{3}\right]-13.6$$

$$\mathrm{SV}\left(\mathrm{mL}\right)=\mathrm{EDV}\left(\mathrm{mL}\right)-\mathrm{ESV}\left(\mathrm{mL}\right)$$

$$\mathrm{CO}\left(L/\min\right)=\frac{\mathrm{SV}\left(\mathrm{mL}\right)\times \mathrm{HR}\left(\mathrm{bpm}\right)}{1000}$$

$$\mathrm{MWTd}(\mathrm{cm})=\frac{\mathrm{LVFWd}+\mathrm{IVSd}}{2}$$

$$\mathrm{RWTd}(\mathrm{cm})=\frac{\mathrm{IVSd}+\mathrm{LVFWd}}{\mathrm{LVIDd}}$$

The remaining cardiac structure and function parameters were derived from the above formulas.

### 2.4. Sample Preparation and Chromatography-Mass Spectrometry Analysis

We followed the broadly targeted metabolomics techniques described in [8,9] and the methodologies reported in previous studies [10,11] with slight modifications. Blood plasma samples were thawed, and 50 µL was mixed with 300 µL of 20% acetonitrile–methanol internal standard extraction solution. The mixture was vortexed for 3 min and centrifuged at 15,000× *g* at 4 °C for 10 min. The supernatants were collected, labeled, and analyzed after standing at 4 °C for 30 min and centrifuging again at 12,000× *g* r/min for 3 min.

#### 2.4.1. UPLC Chromatographic Conditions for T3 Column

Ultra-performance liquid chromatography was performed using a Waters Acquity UPLC HSS T3 C18 column(SCIEX, Framingham, MA, USA), 2.1 mm × 100 mm with 1.8 µm particle size. The mobile phase A was 0.1% formic acid in ultrapure water and phase B was 0.1% formic acid in acetonitrile. The flow rate was 0.4 mL/min, the column temperature was 40 °C, and the injection volume was 2 μL for targeted detection and 5 μL for non-targeted detection.

#### 2.4.2. UPLC Chromatographic Conditions for HILIC Column

UPLC was performed using a Waters Acquity UPLC BEH HILIC column, 1 mm × 100 mm with 1.7 µm particle size; mobile phase A was 20 mM ammonium formate in water with 10% methanol and 60% acetonitrile (pH adjusted to 10.6 with ammonia). The column temperature was 40 °C, the flow rate was 0.4 mL/min, and the injection volume was 2 μL for targeted detection and 5 μL for non-targeted detection.

#### 2.4.3. Mass Spectrometry Conditions for Non-Targeted Detection

Data was acquired by UPLC coupled with a quadrupole time-of-flight (QTOF) triple TOF6600 mass spectrometer ((SCIEX, Framingham, MA, USA)).

#### 2.4.4. Mass Spectrometry Conditions for Broadly Targeted Detection

The data acquisition system consisted of UPLC and tandem mass spectrometry (MS/MS).

For all samples, equal volumes of the extracts were mixed to form quality control (QC) samples. Detection was performed on the LC-QTOF-MS/MS platform, using a self-built standard database (MWDB), a public database (Metlin, HMDB, KEGG), and AI-predicted databases (MetDNA) for accurate qualitative analysis. Data were then extracted, and identified metabolites were subjected to quantitative analysis using Q-Trap instrumentation(SCIEX, Framingham, MA, USA).

### 2.5. Data Processing and Analysis

Cardiac structure parameters were measured using the built-in algorithm of the Mindray M6 veterinary portable color Doppler ultrasound system. The statistical analysis of the cardiac structure indices was performed using SPSS software (version 26.0) with one-way ANOVA, Homogeneity of variance within groups was tested, with *p* > 0.05 indicating no significant difference.

Differential metabolites were selected based on the variable importance in projection (VIP) value from the OPLS-DA model, with a selection criterion of VIP > 1 and *p* < 0.05. Metabolites were annotated using the Kyoto Encyclopedia of Genes and Genomes (KEGG) pathway database [12].

Pearson’s correlation analysis was conducted to analyze the relationship between differential metabolites and cardiac dimensions and function among the three groups, with *p* < 0.05 considered significant.

## 3. Results

### 3.1. Echocardiographic Parameters of Horses

Table 1 presents the cardiac structural and functional parameters of the UG, HG, and AG groups as mean ± standard deviation. As shown in the table, significant differences (*p* < 0.05) were observed between the trained horses and the untrained group in several echocardiographic indices, including LVID, LVFW, LVM, AODd, IVSs, HR, EDV, ESV, LADs, LVLD, MVD, PADs, and SV. Furthermore, comparison between the two trained groups (AG vs. HG) revealed significant differences in AODd, EESV, HR, IVSd, LVIDs, LVM, RVDd, and RVDs (*p* < 0.05).

### 3.2. Broad-Targeted Metabolomics Analysis

#### 3.2.1. Total Metabolite Principal Component Analysis (PCA) and Orthogonal Partial Least Squares-Discriminant Analysis (OPLS-DA)

A total of 1371 metabolites were detected with a UPLC-MS/MS detection platform using a broadly targeted metabolomics approach. PCA was initially performed on all experimental samples and quality control samples to assess the degree of variation between samples (Figure 4A). There was some separation between the AG and HG groups, indicating commonalities in the metabolomes of horses with different exercise regimens. There were good separations between AG and UG, and between HG and UG. All samples were within the elliptical confidence interval, with clear distinctions between different groups. The OPLS-DA results showed that the model was stable and reliable, with no overfitting, and accurately described the samples (Figure 4B).

#### 3.2.2. Differential Metabolite Analysis

**Figure 5:**
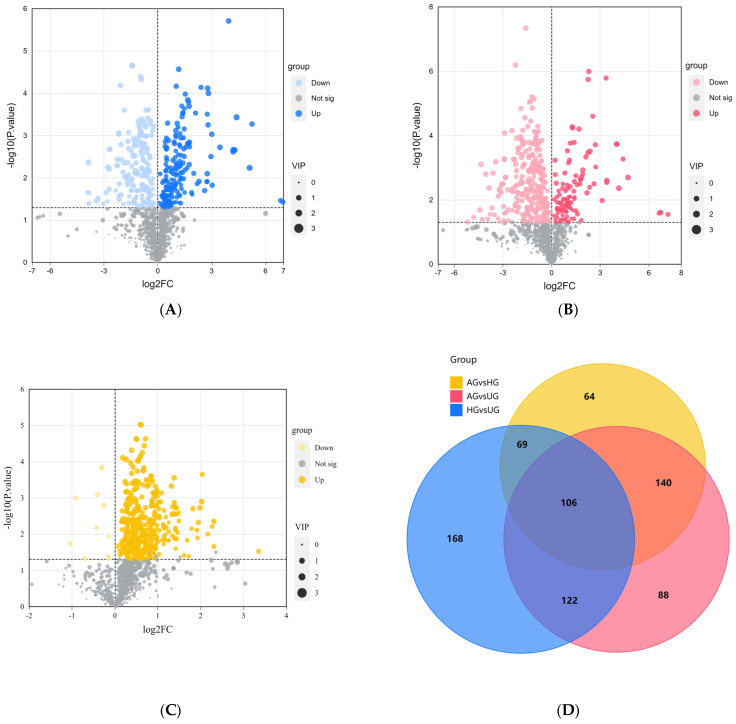
(**A**) HG vs. UG. (**B**) AG vs. UG. (**C**) AG vs. HG. Volcano plots of differential metabolites between groups. (**D**) Venn diagram of shared differential metabolites between AG vs. UG, HG vs. UG and AG vs. HG.

Based on VIP > 1.0 and *p* < 0.05, we identified significantly different metabolites between the groups. The data showed that there were 465 differential metabolites between AG and HG, with 452 upregulated and 13 downregulated. Most of these metabolites were amino acids, followed by benzene derivatives. Between AG and UG, there were 456 differential metabolites, of which 108 were upregulated and 348 downregulated. Most of these were organic acids and derivatives, followed by glycerophospholipids. There were 379 differential metabolites between HG and UG, with 198 upregulated and 181 downregulated. Similarly, most of these metabolites were organic acids and derivatives, followed by amino acids and their metabolites. The volcano plots for the three groups are shown in Figure 5A–C. The differential metabolite relationship between the groups is shown in the Venn diagram (Figure 5D).

#### 3.2.3. Analysis of Shared Differential Metabolites Among the Three Groups

**Figure 6:**
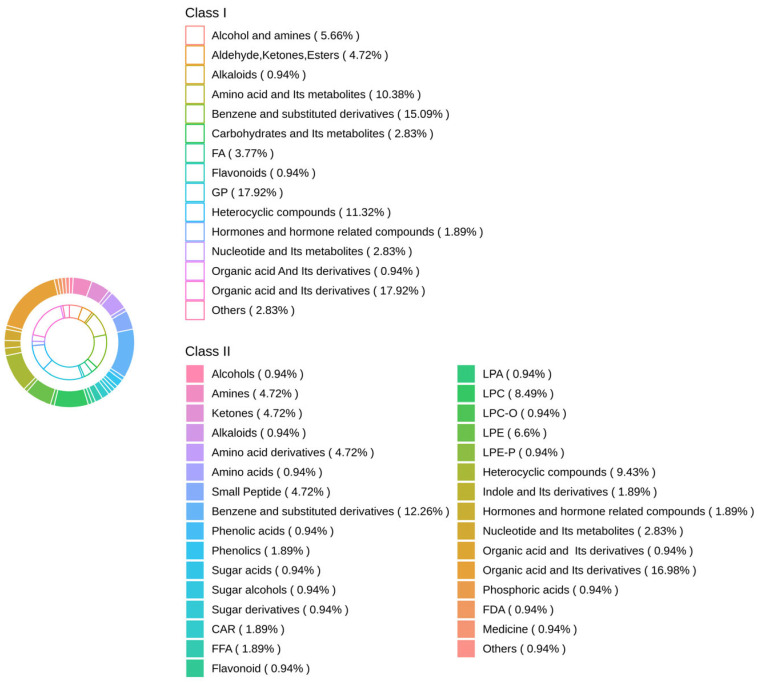
Differential metabolites among the three groups.

Further analysis revealed 106 shared differential metabolites among the three groups, and their classification information is shown in Figure 6. There were significant differences among the three groups with organic acid and derivatives being the most prevalent metabolite, GP being the second, and benzene and substituted derivatives being the third.

#### 3.2.4. KEGG Pathway Enrichment Analysis of Differential Metabolites

**Figure 7:**
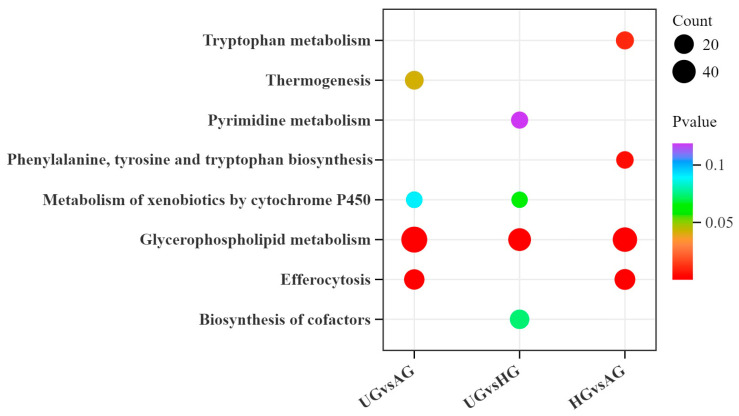
KEGG bubble chart of differential metabolite enrichment between the three exercise groups.

KEGG pathway enrichment analysis was performed based on the results of the differential metabolite analysis. The differential metabolites between AG and HG were mainly enriched in bile secretion, cofactor synthesis, and metabolic pathways. Between AG and UG, the differential metabolites were primarily enriched in glycerophospholipid metabolism, choline metabolism in cancer, and the one-carbon pool by the folate pathway. Between HG and UG, they were mainly enriched in pathways of glycerophospholipid metabolism, choline metabolism in cancer, and biosynthesis of unsaturated fatty acids. Thirty-seven metabolic pathways overlapped across the comparison groups, including 2-oxocarboxylic acid metabolism, arachidonic acid metabolism, and fatty acid degradation (Figure 7).

**Figure 8:**
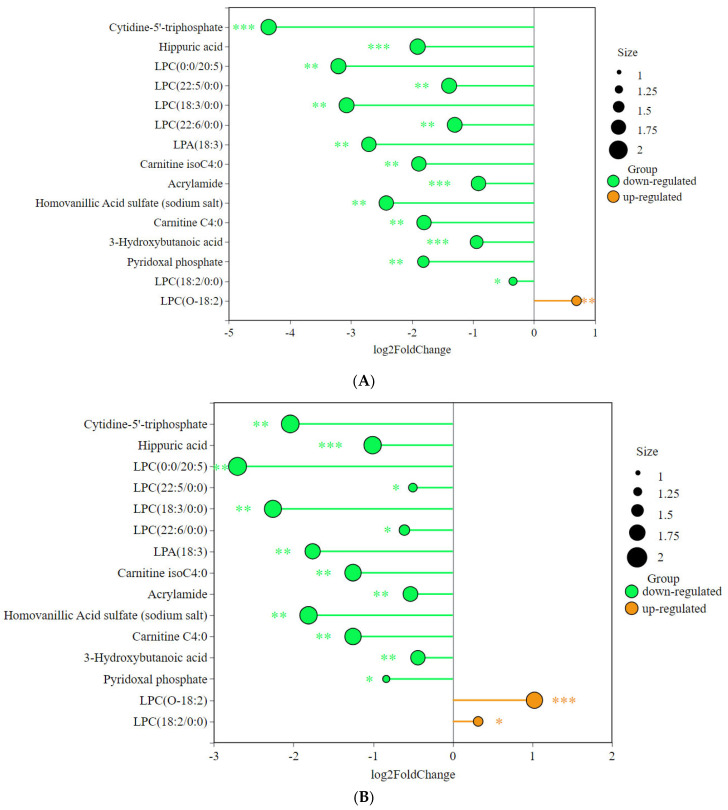

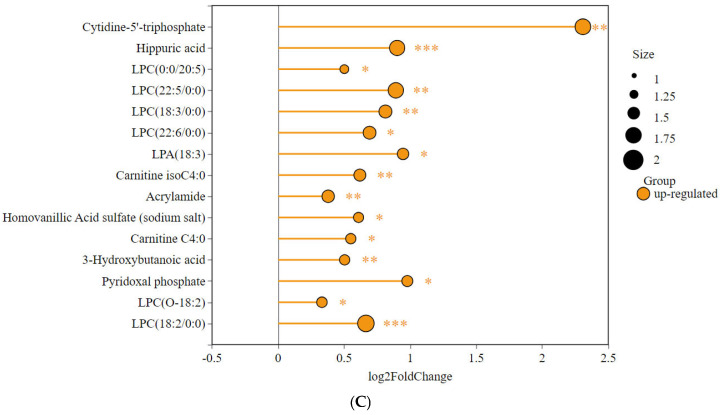
Differences in abundance of key metabolites among the three groups: (**A**) AG vs. UG, (**B**) HG vs. UG, and (**C**) HG vs. AG. (* *p* < 0.05, ** *p* < 0.01, *** *p* < 0.001).

We found 15 significantly different metabolites relevant to this study (Figure 8). The graph shows that the advanced group and the habitual group have several metabolites in common compared to the untrained group.

#### 3.2.5. Correlation Analysis Between Differential Metabolites and Cardiac Structures

**Figure 9:**
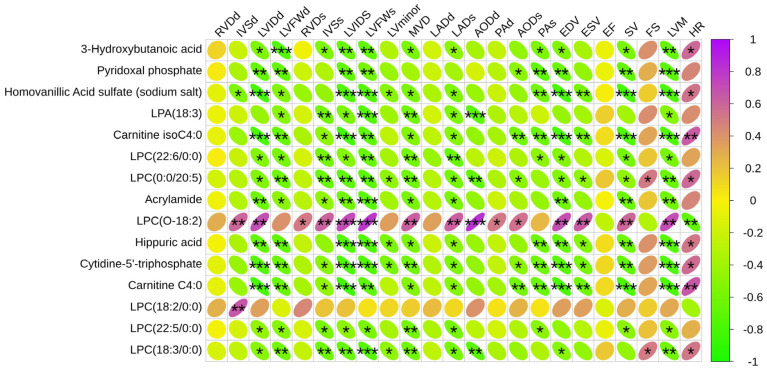
Correlation analysis between significantly different metabolites and structural function of the equine heart. Green is a negative correlation, red is a positive correlation, and the darker the color, the stronger the correlation. * *p* < 0.05, ** *p* < 0.01, *** *p* < 0.001.

The 15 significantly different metabolites were analyzed in conjunction with various cardiac structures to identify the metabolites most closely related to exercise-induced heart remodeling (Figure 9).

## 4. Discussion

The heart is one of the most important organs in the body, and its functional state directly affects the athletic performance of horses, making it a key factor in determining race outcomes [13]. By combining echocardiography and plasma metabolomics, this study effectively identified cardiac biomarkers associated with athletic performance, offering valuable insights into selecting and breeding high-performance horses, improving race results, and promoting the science of equine sports. Baseline echocardiographic and metabolomic data prior to training were not collected; thus, we cannot conclude any predictive power for performance before training. Results reflect post-training differences.

The term ‘athlete’s heart’ refers to the adaptive changes in heart structure and function that occur with mid- to long-term physical training (6 mos). One of the most noticeable changes is a lower resting heart rate. Numerous studies have shown that regular exercise can increase cardiovascular diameters, improve cardiac output, and raise blood pressure, enabling lower heart rates to meet the body’s respiratory needs [14]. In this study, after six months of training, both AG and HG groups had significantly lower heart rates than the UG, indicating that training induced physiological cardiac remodeling in the HG and AG groups.

There are few studies on RVDd and RVDs in horses [15]. In a comparative study of professional athletes engaged in cycling, football, basketball, and wrestling versus a sedentary group, only 35% of athletes showed structural changes in the right ventricle, and these changes were not significant [16]. In this study, no significant differences in right ventricular measurements were found among the three groups, suggesting the need for further research on Yili horses involved in various equestrian disciplines. The IVS at end-diastole and end-systole in the HG and AG groups was significantly greater than in the UG, indicating that high-intensity exercise had a significant structural impact on the heart’s septa. A thicker septum can enhance the heart’s pumping ability [17], which may explain why high-performance horses had thicker septa compared with untrained horses. The LV plays a crucial role in pumping blood, and, during exercise, the LV responds to the body’s increased need for oxygen and nutrients by increasing cardiac output. Mid- to long-term exercise significantly enlarged the LVID, with the HG horses showing significantly larger diameters than the AG and UG horses during both end-diastole and end-systole. Although the differences between AG and UG in LVIDd were not statistically significant, average values showed a trend of increasing diameter with higher exercise intensity. Cardiac mass is influenced by various factors, and mid- to long-term training can lead to an increase in the size of myocardial cells, causing the heart to enlarge as well [18]. For untrained horses, the main factors affecting heart structure were related to growth, and the degree of change was relatively small.

Exercise training significantly affects fatty acid metabolism, amino acid metabolism, and oxidative stress, all of which have notable effects on cardiac structure and function. As shown in Figure 8, many of the differential metabolites were significantly correlated with cardiac structure. LPCs are monoacyl phospholipids produced by hydrolysis of phosphatidylcholine (PC) by phospholipase A2 (PLA2). Lysophosphatidylcholine (LPC) and lysophosphatidic acid (LPA) are the key phospholipids in the synthesis of cardiolipin, which is an essential component of the mitochondrial membrane. Several studies have shown that LPCs are closely related to mitochondrial function, and the LPC content in plasma can be used as a biomarker of mitochondrial function in skeletal muscle, with lower concentrations of specific long-chain LPCs (≥12 carbons) positively correlating with lower mitochondrial function in skeletal muscle [19,20]. This may be because changes in the levels of LPCs affect the structure and function of the inner mitochondrial membrane, which in turn indirectly affects the energy metabolism capacity of mitochondria. For example, decreased levels of LPCs may lead to changes in the curvature of the mitochondrial inner membrane, which in turn affects the efficiency of the electron transport chain [21]. LPC (22:6/0:0), LPC (0:0/20:5), LPC (18:2/0:0), and LPC (22:5/0:0) were significantly negatively correlated with LVIDd, LVFWD, LVIDs, LVLD, MVD, and LADs in this study. This may be due to the higher mitochondrial function and fatty acid oxidizing capacity in athletic horses. Due to anatomical complexity and limits on ultrasound resolution, measurements of LVLD and MVD have inherent inaccuracies. The data should be interpreted as relative changes, not absolute reference values. As an intermediate, LPC may be broken down for energy production into other products such as phosphatidic acid through β-oxidation. Prolonged exercise also enhances the activity of lipoprotein-associated phospholipase (Lp-PLA2) and acyltransferase, which accelerates the metabolism of LPC, leading to a decrease in circulating LPC concentration. Of interest, LPC (O-18:2) showed a significant positive correlation with some cardiac structures and functions and a significant negative correlation with heart rate, contrary to the pattern for other LPCs. The ether or ester bonds that link fatty acid chains to the glycerol backbone are specific chemical linkages that differ significantly in structure, stability, and biological function. The ether-bonded phospholipids are more stable and are mainly found in specific tissues; their location and function should be further investigated as a biomarker.

3-Hydroxybutanoic acid is a ketone body that is normally generated in liver mitochondria by β-oxidation of fatty acids [22], which provides energy to the brain, heart, and skeletal muscle when glucose levels are not sufficient. In the present study, its abundance was lowest in AG, moderate in HG, and highest in UG. This may be because prolonged exercise training significantly increases the fatty acid oxidation capacity of skeletal muscle and enhances mitochondrial β-oxidation efficiency. This adaptation makes athletic individuals more inclined to directly oxidize free fatty acids (FFAs) [23] rather than relying on the liver to convert them to ketone bodies through ketogenesis [24]. Endurance training induces an increase in the activity of enzymes related to ketone body metabolism in muscle and promotes the rapid utilization of ketone bodies as fuel [25]. Carnitine C4:0 and carnitine isoC4:0 belong to the family of carnitines. Carnitine C4:0 optimizes the balance between beta-oxidation and glucose metabolism by scavenging excess short- and medium-chain acyl-coenzyme A (acyl-CoA) from mitochondria and by maintaining the concentration of free coenzyme A (CoASH). In skeletal and cardiac muscle, carnitine C4:0 promotes glucose oxidation by modulating the acetyl-CoA/CoA ratio and relieving pyruvate dehydrogenase complex (PDC) inhibition [26]. Carnitine isoC4:0 is a branched isomer of carnitine C4:0 with a different metabolic pathway from carnitine C4:0. Carnitine isoC4:0 has been demonstrated to be useful not only as a diagnostic and predictive biomarker of heart failure but also for determining the primary etiology of heart failure and prognosis of the disease [27]. In the present study, these two carnitines showed the same pattern of change as 3-hydroxybutanoic acid, which may be due to the fact that short-chain acylcarnitines are carriers of fatty acid oxidation intermediates, and their concentrations may reflect metabolic stress or substrate utilization efficiency; this needs to be tested in further studies.

The pattern is similar to that of 3-hydroxybutanoic acid, in which the concentration was lowest in the AG, at a medium level in the HG, and highest in the UG. Hippuric acid, a product of phenylalanine metabolism, can accumulate as the intensity and duration of exercise increase [28]. The metabolism of amino acids, such as phenylalanine, is enhanced during exercise to meet the body’s energy needs. The results of a study of plasma metabolites in Mongolian horses after a long-distance endurance race showed a significant increase in uric acid and a decrease in methanol, suggesting that the gut microbial activity of the horse was suppressed during exercise [29,30]. Another study found that the concentration of uric acid in horses was significantly and negatively correlated with peak myocardial power and aerobic exercise capacity, suggesting that equine uric acid metabolism may have an effect on cardiac function [31]. In this study, compared with the non-trained group, the level of equine uric acid was lower in the trained groups, and the abundance in the AG group was lower than that in the HG group, which is similar to the results of the previous study in which the rate of equine uric acid metabolism was higher in horses with mid- to long-term training. Equine uric acid was also significantly negatively correlated with LVID, LVM, and this finding should be further investigated as a sports performance marker.

In this study, pathway enrichment analysis of resting plasma metabolomes reveals different metabolic adaptations between horses with different exercise capacities. Notably, glycerophospholipid metabolism was the core pathway (significantly enriched among all three groups) that differentiated exercise performance. Glycerophospholipids are major components of cell membranes and important signaling mediators [32]. The differences may reflect the dynamic remodeling of cell membranes by training in horses of the AG group. Differential metabolite enrichment in the efferocytosis pathway was observed in the AG versus HG and UG. Cell damage and apoptosis are inevitable during exercise, and effective apoptotic cell clearance mechanisms are essential for maintaining tissue homeostasis and promoting recovery. A recent study showed that regular exercise can promote tissue repair through modulation of macrophage function [33]. AG horses may maintain tissue homeostasis and accelerate the recovery process through this mechanism. Differential metabolites of AG versus HG were significantly enriched in tryptophan metabolism and phenylalanine, tyrosine, and tryptophan biosynthesis. Tryptophan metabolism has an important role in the regulation of immune responses, neurotransmission, and energy production [34], suggesting strong synergistic regulation of neuro-endocrine-immune networks in athletic horses.

## 5. Conclusions

The plasma metabolome of horses with different athletic performance after mid- to long-term conditioning and training demonstrated significant correlations with changes in the structure and function of the heart, including significant differences in left ventricular end-systolic internal diameter, left ventricular mass, and heart rate ESV. Changes in plasma levels of LPC, 3-hydroxybutanoic acid, carnitine C4:0, and carnitine isoC4:0 can be used as discriminatory indicators of exercise performance in horses. The metabolic pathways involved in the differential metabolites produced by horses after mid- to long-term training mainly involve glycerophospholipid metabolism. Equine plasma metabolomics analysis has excellent potential for monitoring and evaluating equine athletic status.

## Figures and Tables

**Table 1:** Cardiac structure parameters for the three groups of Yili horses.

Parameter	UG	HG	AG
RVDd (cm)	2.98 ± 0.15 ^ab^	2.91 ± 0.47 ^b^	3.32 ± 0.29 ^a^
IVSd (cm)	2.46 ± 0.15 ^b^	2.55 ± 0.12 ^b^	2.86 ± 0.15 ^a^
LVIDd (cm)	9.69 ± 0.35 ^b^	10.24 ± 0.12 ^a^	10.51 ± 0.19 ^a^
LVFWd (cm)	1.99 ± 0.18 ^b^	2.47 ± 0.35 ^a^	2.38 ± 0.21 ^a^
RVDs (cm)	2 ± 0.18 ^b^	2.1 ± 0.32 ^b^	2.5 ± 0.25 ^a^
IVSs (cm)	3.82 ± 0.17 ^b^	4.28 ± 0.26 ^a^	4.42 ± 0.41 ^a^
LVIDS (cm)	4.94 ± 0.18 ^c^	5.31 ± 0.13 ^b^	5.48 ± 0.08 ^a^
LVFWs (cm)	2.82 ± 0.18 ^b^	3.61 ± 0.24 ^a^	3.67 ± 0.31 ^a^
LVLD (cm)	14.98 ± 0.44 ^a^	15.42 ± 0.37 ^a^	15.56 ± 0.61 ^a^
MVD (cm)	8.49 ± 0.11 ^b^	9.17 ± 0.39 ^a^	9.45 ± 0.54 ^a^
LADd (cm)	8.89 ± 0.44 ^b^	9.15 ± 0.77 ^ab^	9.75 ± 0.78 ^a^
LADs (cm)	9.79 ± 0.39 ^b^	10.67 ± 0.49 ^a^	10.99 ± 0.71 ^a^
AODd (cm)	5.27 ± 0.23 ^c^	5.63 ± 0.19 ^b^	5.95 ± 0.18 ^a^
PADd (cm)	4.28 ± 0.22 ^a^	4.52 ± 0.33 ^a^	4.47 ± 0.2 ^a^
AODs (cm)	5.56 ± 0.24 ^b^	5.81 ± 0.32 ^ab^	5.97 ± 0.03 ^a^
PADs (cm)	4.85 ± 0.36 ^b^	5.25 ± 0.37 ^a^	5.27 ± 0.21 ^a^
EDV (mL)	527.72 ± 42.92 ^b^	594.51 ± 15.36 ^a^	630.12 ± 24.82 ^a^
ESV (mL)	193.29 ± 23.9 ^c^	215.57 ± 12.73 ^b^	241.09 ± 14.9 ^a^
EF (%)	0.63 ± 0.03 ^a^	0.64 ± 0.02 ^a^	0.62 ± 0.02 ^a^
SV (mL)	334.43 ± 28.26 ^b^	378.94 ± 17.29 ^a^	389.03 ± 21.22 ^a^
FS (%)	49.03 ± 1.2 ^a^	48.16 ± 1.36 ^a^	47.89 ± 1.17 ^a^
LVM (g)	1985.72 ± 223.05 ^c^	2568.44 ± 282.02 ^b^	2841.5 ± 103.67 ^a^
HR (bpm)	45 ^a^	40 ^b^	39 ^c^

Notes: UG, untrained group; AG, advanced group; HG, habitual group. Statistical comparisons of echocardiography results from the three performance groups of Yili horses. Comparison of cardiac parameters among the three groups. Different letters labeling the columns mean that a certain value is significantly different in the two groups. If the letters are the same, it means that there is no significant difference (the letters themselves have no special meaning). IVSd, end-diastolic interventricular septal thickness; LVIDd, end-diastolic left ventricular diameter; LVFWd, end-diastolic left ventricular free wall thickness; RVDs, end-systolic right ventricular diameter; IVSs, end-systolic interventricular septal thickness; LVIDs, end-systolic left ventricular diameter; LVFWs, end-systolic left ventricular free wall thickness; LVLD, left ventricle long axis diameter; MVD, mitral valve diameter; LADd, end-diastolic left atrial diameter; LADs, end-systolic left atrial diameter; AODd, end-diastolic aortic root diameter; PADd, end-diastolic pulmonary artery diameter; AODs, end-systolic aortic root diameter; PADs, end-systolic pulmonary artery diameter; EDV, end-diastolic left ventricular volume; ESV, end-systolic left ventricular volume; EF, ejection fraction; SV, stroke volume; FS, fractional shortening; LVM, left ventricular myocardial mass; HR, heart rate.

## Data Availability

The original contributions presented in the study are included in the article/Supplementary Materials, further inquiries can be directed to the corresponding authors.

## Supplementary Materials

The following supporting information can be downloaded at: https://www.mdpi.com/article/10.3390/ani15162444/s1, Table S1. Yili Horse Size Data; Table S2. Yili Horse 1000 m Speed Race Results; Table S3. Parameters of cardiac structure and function of untrained horses; Supplementary Text S1: Training plan.
