# Supplementary material for: Differential Metabolomics and Cardiac Function in Trained vs. Untrained Yili Performance Horses
Source: Animals (Basel). 2025 Aug 20;15(16):2444. doi: 10.3390/ani15162444 (PMC12382719; doi:10.3390/ani15162444)
Supplement: Supplementary file 1 [file animals-15-02444-s001.zip › animals-3734115 Supplementary Text S1 Training plan.pdf]

Supplementary Text S1

Training Plan

The conditioning schedule for the horses was as follows:
Week 1: Round pen groqundwork (sand), back adaptation exercises, mounting in the stable, and leading practice.
Week 2: Warm-up in the round pen and on the mechanical walker for 40 min, followed by 20 min of riding.
Week 3: Similar warm-up, followed by 20 min of riding and practicing fixed tracks, including serpentine, figure-eight, Z-shaped, and circular routes.
Week 4: Round pen groundwork(sand), 35 min of warm-up on the mechanical walker, and 25 min of riding. Training included slow walking (800 m), trotting (800 m), and slow walking (800 m) on the track(sand).
Week 5: Trotting distance was increased to 1600 m, while the other activities remained same with Week 4.
Week 6: Round pen groundwork and mechanical walker warm-up for 30 min, followed by 30 min of riding. Training included short sprints (400 m) and trotting (800 m). For the next five days, sprint distance was increased by 50 m daily, while the trotting distance was reduced by 50 m.
Week 7: Warm-up, 30 min of riding, 800 m trotting, 800 m short sprints, and 800 m slow walking.
Week 8: Warm-up as in Week 7, followed by 1200 m short sprints on sand.
Week 9: Similar warm-up and track training as Week 8, with grass sprints increased to 2100 m.
Week 10: Warm-up, 30 min of riding, 800 m slow walking, 800 m trotting, and gradual acceleration on grass until reaching a gallop.

At 9:00 AM, all horses were moved to two paddocks (sand): the training group was placed in one paddock and subsequently underwent the training program on that day, while the non-training group was placed in the other paddock for free movement. During intervals between training sessions, horses from both groups were allowed free movement in the paddocks (sand). At 9:00 PM, all horses were brought back into the stables.

Concerning the high intensity of the training program, we designed a progressive schedule: during the first month, horses were trained at 60% of the planned intensity; during the following two months, at 80% (with the content of the first week skipped); and during the final three months, at 100% intensity (also with the content of the first week skipped). Heart rate monitors were used to assess exercise intensity, with HRmax set at 240 bpm. Training was conducted five days per week, with daily training duration strictly adhering to the program outline.

The training plan was designed based on heart rate zones: trotting at 50–60% HRmax, galloping at 70% HRmax, and slow walking. Riding and track training sessions were implemented accordingly. The six horses in the UN group were not subjected to training and were allowed free movement in the paddock (sand). At the end of the training period, the performance of the horses was tested by a 1000 m race. Based on the results, the top six performers were assigned to the AG group (average time: 75.7±1.56 s), and the lower six performers were assigned to the HG group (average time: 80.24 ± 2.36 s).
